# Michigan State Dental Association

**Published:** 1859-02

**Authors:** 


### MICHIGAN STATE DENTAL ASSOCIATION.

Fourth Annual Meeting, held in the city of Detroit, January, 11th and 12th, 1859.

Agreeable to published notice, the Society met at the office of Drs. Whiting & Benedict, at 7 o'clock, P. M.

Both President and Vice-President being absent--

Dr. R. S. Bancroft, of Romeo, was appointed chairman, and John A. Harris, of Pontiac, secretary, pro tem.

The minutes of proceedings of last meeting were read and approved.

Letters were read from the President and Vice-President and several members, stating the cause of absence, and expressing regrets therefor, with sentiments of sympathy and encouragement.

The Society now proceeded to the election of officers for the ensuing year.

Dr. C. B. Porter, of Ann Arbor, was elected President.

Dr. R. S. Bancroft, of Romeo, Vice-President.

Dr. L. C. Whiting, of Detroit, Secretary and Treasurer.

No special order of business having been prepared, it was resolved by acclamation, to take up the same subjects for discussion, and in the same order, as at the previous meeting.

The election to membership was now proceeded with. Six new members were added to the list, and on motion of Dr. Harris, of Pontiac,

John T. Toland, from Cincinnati, was elected as an honorary member, by acclamation.

Members Present.—Drs. H. Benedict, L. C. Whiting, W. Cohoon, C. F. Knowlton, Geo. L. Fields, T. A. White, J. H. Farmer, R. V. Ashley, D. C. Prevost, of Detroit; R. S. Bancroft, from Romeo; T. D. Ingersoll, Monroe; C. B. Porter, Ann Arbor; John A. Harris, Pontiac; A. F. Barr, Ypsilanti; S. C. Smith, Flint; C. S. Chittenden, Hamilton, C. W.; John T. Toland, Cincinnati, O. Besides which, quite a number of gentlemen, dentists and physicians were in attendance, the greater portion of the time, making an assembly of from twenty to twenty-five persons.

The discussions were now opened, each member being limited to ten minutes.

### Subject 1st.—Preparing Cavities.

From the difficulty of illustrating, and the fact that nearly every case requires different treatment, calling into aid the judgment, tact and skill of the operator, there was a manifest reluctance to open the discussion on this subject.

Dr. Whiting, however, gave a very interesting explanation of his mode in a variety of cases, filing, wedging, etc., as the case requires, dependent on the situation of the teeth, age of the patient, etc.

Several others made some remarks, more in the form of conversation than discussion, all of which was deeply interesting and gratifying.

Adjourned to meet at same place to-morrow morning at nine o'clock.

WEDNESDAY MORNING, Jan. 12th, 9 o'clock.

Society met according to adjournment—minutes read and approved.

SUBJECT 2ND,—FILLING TEETH,

Was taken up and the discussion opened by

Dr. Benedict, who gave his experience and practice in different cavities, and different materials used, both foil and crystal gold, in some cases prefers foil, in others sponge. In reply to question, stated how he treats sensitive dentine, abscess, etc., to the satisfaction of all present.

Dr. White, in shallow cavities of the front teeth with wall broken, finds it frequently difficult to get a good start, especially when the decay extends near to the nerve, is very careful not to expose or puncture the nerve, uses creosote, arnica and origanum, to allay sensitiveness, then varnishes the cavity with gutta percha, which being a non-conductor protects the pulp from the sudden changes of temperature, chisels down the ragged edges to obtain smooth strong edges, thinks the most of failures occur from a neglect of this precaution, tries to get points dovetailed, if there are no points he makes them, sometimes cuts a groove around the inside margin. Uses both foil and sponge but lately crystal gold alone, having dryed the cavity, (the dryer the better) he proceeds to fill, pressing lightly at first, fastening the gold in retaining points, and building a bridge over the nerve, and thus continues adding layer after layer until the cavity is full. In this way he has always been successful.

Dr. Chittenden don't coincide with all the ideas advanced, not met with much success in wedging, but still uses it. Take the case of a central incisor, with a large approximate cavity, first cut an opening with a chisel, then with a half round or oval file, separates well, so as to give a strong wall and enable him to prepare the cavity well. Then, with serrated instruments and soft foil (generally Abbey's) in pellets, begins at the bottom of the cavity, and with hard pressure packs well up to the bottom and toward the gum. After a good foundation is laid, then takes crystal gold foil and fill up flush, pierce a hole in the center and pack in a wedge, the object being not so much to make it adhere, as to have the cavity perfectly full, it don't do to anneal foil in pellets, as it makes a solid mass, he prefers always to have a strong wall, and generally cuts away until it is strong enough to resist all the force he can apply. The Doctor exhibited his mode of forming pellets, which is the same as the cylinder, described by numerous writers. A slate and pencil were now procured, and the discussion run into a conversational and illustrative character, though highly instructive, is impossible to report.

The constitution was now read by the secretary and signed by new members, initiation fees paid, etc.

On motion the rules were suspended, and the remainder of the forenoon was occupied in relating cases of interest which have fallen under the notice of different members.

Dr. Chittenden related the case of a child, kicked by a horse on the chin, process fractured, some of the teeth lost, some buried in the process and afterwards resumed their proper place without any scientific aid.

Dr. Harris. A case of fracture of process involving three molars and dens sapientia teeth, occurred in the attempt by a physician to extract a second bicuspid, dissected around and removed the process with teeth attached.

Dr. Smith. A case of gradual displacement of an incisor tooth, caused by the force of the lower teeth striking against it, until it protruded to the annoyance of the patient, and was extracted. Approved.

Dr White. Case of diseased antrum, great pain, inflammation, physician advised removal of the jaw. On examination by self and Dr. Cohoon, opinions concurring, removed the roots of two bicuspids, opened the antrum, from which a large quantity of offensive matter was discharged, treated with creosote and perfectly restored.

Dr. Whiting treated a scar in the cheek, called by a physician cancer. Removed an offensive tooth, healed the sore and removed the depression by adapting a piece of tin with Burgundy pitch, to draw it to the proper shape.

Dr. Smith. Similar case cured by pledgets of cotton between cheek and tooth, saturated in tinct. arnica, and renewed daily.

Dr. Field. A case—hole in gum size of wire excavator, considerable discharge and no pain. The fang of a bicuspid transverse to the opening. Asked advice, which was given, viz: to remove fang and treat with camphor and creosote.

Dr. Chittenden, called by physician in consultation, to remove necrosed bone, introduced a probe and out popped a fang, discharge ceased and man got well.

Dr. Harris. Case—teeth badly decayed, patient in picking them with a pin, the pin slipped out of his fingers and disappeared. Probed about two inches but failed to find it. No pain.

Dr. Field also exhibited a second molar and dens sapientia, the posterior fang of molar perfectly united to the anterior surface of the dens sepientia, so as to form an angle of thirty-five to forty degrees.*

In bleeding, after the extracting of teeth, Dr. Harris uses tannin and ether.

Dr. Field uses myrrh and camphor, on cotton held by a piece of cork and bandage.

Dr. Farmer, perchloride of iron, and related an interesting illustration of its effects, for which we have not room.

Dr. Chittenden thinks, in protracted bleeding, the gums are also implicated, has used tinc. perchloride of iron with never-failing success.

Dr. White has but one panacea, strong vinegar with alum, and compress.

On motion, adjourned to two o'clock.

AFTERNOON SESSION, WEDNESDAY, 2 O'CLOCK.

Society met pursuant to adjournment. On motion, reading of minutes was dispensed with.

Several bills were presented, for expenses incurred since last meeting, all of which, on examination, were approved and ordered to be paid.

### SUBJECT 3RD.—TREATMENT OF EXPOSED NERVES.

The discussion was opened by the reading of a paper from Dr. Mansfield, on this subject, which will be found in another part of the REPORTER, under the head of Original Communications.

Dr. Whiting has treated nerves and filled fangs, as described by Dr. Mansfield, frequently finds deposits of Lime at the point of the root, and thinks it necessary that such deposits be removed in order to ensure a healthy condition, sometimes the nerve cavity is compressed, he considers it necessary to open the cavity, so as to get to the point of the fang, by some means,

*Dr. F. has promised to give us the history and description of this case in detail, with an engraving of the teeth in position, for the next number of Reporter. And we would suggest that each of the gentlemen under whose observation the foregoing cases occurred, will do likewise. These are cases of much interest, and it is from such that facts are deduced, and great scientific principles established.

remove the deposit, and by careful treatment the fang will become healthy.

Dr. Ingersoll does not claim anything new. In these cases it is necessary to labor both mentally and physically, the chief object is to get the fang perfectly clean and healthy, and to induce his patient to exercise patience. To do this, he places the patients on their own responsibility which causes them to be careful in observing instructions, in this way he is enabled to save a great many. Has treated ulcerated fangs, where he could pass a probe beyond the point of the root, with care and patience has succeeded.

Dr. Chittenden has been doing fang filling only about a year, and to his surprise has lost none. Never attempts molars, because he can not be certain of the fangs being perfectly clean. Never observed any appearance of calcarious deposit. Uses for destroying nerves, equal parts of arsenic and morphine with creosote, generally allows it to remain twenty-four hours, sometimes has to repeat, has by neglect of patient remained a week without any appearance of injury.

Dr. Bancroft uses arsenic and pledget of cotton saturated with creosote, retains application from six to twenty-four hours, if convenient to see the patient in six hours, and finds the work accomplished, he removes the application. He finds less complaint of pain from this application than when arsenic and morphine are combined.

Dr. Smith has used chloroform, and thinks it better than morphia, does not use it in any specific quantity, but saturates a piece of cotton, places it in the cavity in contact with the exposed pulp, and covers it *quickly* with wax, and has no trouble, rarely fills in less than a week or ten days after destroying the nerve, the chloroform destroys the nerve.

Dr. White is rather inclined to stick to the old style at the expense of being considered an "old fogy," has used equal parts arsenic and morphia, mixed in conserve rosa, but now uses arsenic alone on a pledget of cotton, saturated with creosote and rarely finds more than one application necessary. If there seems to be any degree of inflammation takes a small pledget of cotton saturated with creosote, passes it to the end of the fang and lets it remain there, fills immediately and finds less trouble than by waiting.

Dr. Farmer uses three parts arsenic and two parts sulph. morp., which rarely produces pain, when it does you have only to wait the proper length of time and it will subside. Has met with cases where the nerve seemed to be destroyed, but great tenderness existed at the apex of fang, in such cases has repeated the operation with satisfactory results.

Dr. Porter's expirience is similar to the foregoing. He has met with cases of tenderness of the apex when the nerve seemed to be destroyed, but on close scrutiny has discovered in such cases a *film* of bone to obstruct the nerve canal, and cut off communication and prevent the medicine from being conducted to that part of the nerve beyond this barrier. If this bony film be removed and the application renewed, there can be no difficulty. Prefers to remove the application as early as possible, if allowed to remain too long the arsenic is absorbed by the bone, which produces a soreness or sensitiveness of dentine, which it is difficult to remove.

Dr. Benedict here related a little experience. A lady called to have a tooth mounted on pivot, the fang was very sore, after considerable questioning he discovered that she had been trying to kill the nerve with arsenic. She had evidently applied too much, and the bone had absorbed it, he applied creasote, closed the cavity and let it remain ten days, when all soreness had disappeared, he filled the fang, mounted a tooth and had no further trouble.

SUBJECT 4TH. ALVEOLAR ABSCESS.

Dr. Benedict opened the discussion. His method of treatment consists in drilling, or otherwise cutting through, so as to pass a probe or broach in freely, then treat by application of creosote or nitrate of silver.

Dr. Chittenden pursues the same course, except that he used nitrate of silver first, to cleanse the cavity.

Dr. Whiting proceeds the same as in the treatment of a diseased fang, cleanses and applies creosote, rarely has any trouble, but sometimes when the patient takes cold the symptoms return.

Dr. Smith thinks there is but little use of any kind of treatment. If the tooth is *dead* there must be a discharge, and unless the absorbents *take it* up, *it* must make its escape, therefore seldom attempts to treat at all.

Dr. Bancroft's experience is similar to Dr. Smith's, but with a few exceptions of successful cases.

Dr. Whiting agrees with the two last speakers. When the periosteum is destroyed and all the attachments broken up, there is but little hope of success, has tried all modes recommended, has cut, scraped, etc., seeking for the bottom, but with no great success.

Dr. Porter reported a very hard case of a *central incisor* in which he tried nit. argart, chlo. soda. creosote, etc., and treated it nearly six months without success, finally used chloride of zinc, which healed it promptly, after which he filled the fang and crown with gold, and it is now doing well—this occured several years ago. Nearly all incisors and cuspidati succeed, but bicuspids and molars are doubtful.

SUBJECT 5TH.—MECHANICAL DENTISTRY.

Dr. Chittenden leads the discussion. He takes impressions in wax, and uses cups of peculiar construction, viz: with the center raised as high as the marginal edge of the cup, with a projecting *flange* on the outside to press out the cheeks and lips to prevent it from dragging in removing from the mouth. Uses plaster in some special cases of upper sets.

Dr. Benedict uses plaster both above and below for full sets, mixed about as thick as cream, stirring constantly until *nearly* ready to *set*, then places it in the mouth and retains it immovably until it is set, which requires from 5 to 8 minutes.

Dr. Smith uses wax, tried plaster without success.

Dr. Whiting. 'Although the taking of impressions is of great importance, it is not all—it is quite as important to obtain a good cast from the impression; thinks best to varnish the plaster casts with shellac, then oil, which causes them to separate readily, does not observe any specific time, but puts it through as fast as possible. and had the best success when most hurried.

Dr. Bancroft thinks it important to take casts as quickly as possible, from plaster impressions, otherwise the expansion of the plaster will destroy the fit.

Dr. White thinks plaster expands for the first twenty-four hours and afterwards contracts, and recommends rapid manipulation. The greatest difficulty occurs in partial sets, where the teeth are irregular. In such cases finds *gutta percha* just the thing. Warm it and press firmly to the arch, sometimes there is a little difficulty in removing, but its elasticity enables the operator to remove it, and when removed, it resumes the position it held in the mouth.

Dr. Farmer mixes wax and resin, fifteen per cent. of the latter. Sometimes uses gutta percha, and with Dr. White, thinks it excellent he warms it in hot water until sufficiently soft, and is enabled to take an impression so *fine* as to preserve the ridges and irregularities on the natural teeth.

On motion, adjourned until 7 o'clock, P. M.

EVENING SESSION.

Society met agreeable to adjournment, at seven o'clock, taking up the—

### Subject 6th.—Getting up Casts.

Dr. Whiting, leading off in good style—says after taking plaster cast, he takes an accurate model in sand, for male dies he uses zinc, sometimes adding antimony, (but after a few meltings the antimony is lost,) which he pours into the model of sand, then melts a quantity of lead and dips the male cast in, holding it steadily until the lead hardens by cooling, but when a particularly nice cast is wanted, he places the male cast in a box and pours the lead upon it, and in difficult and irregular cases, uses Howe's Flask.

Dr. Bancroft's method is similar to the above, but sometimes uses one-third type metal which mixed with the other metals, makes the casts harder and requires a less number of duplicates.

Dr. Chittenden's mode differs but slightly from Dr. W. Ordinary zinc leaves little nasty indentations in the plate. Now uses sheet zinc, which is more smooth and uniform, but in very particular cases uses pure tin and lead.

Dr. Benedict thinks if very fine moulding sand is used and the metal not too hot, no difference will be found between *slab* and *sheet* zinc, and no difficulty with either.

Dr. Fields generally uses *sheet* zinc for male casts, but in very particular cases uses brass, it being harder and smoother.

### Subject 7th.—Mounting Artificial Teeth.

Dr. Benedict, with great minuteness, describes his mode as follows:

Takes the impressions and casts as previously described—uses gold plate, *never less* than nineteen carats fine, swage plates between zinc and lead, not too much at first, uses a second set of casts and brings up very near as close as wanted, turns the edges of the rim, takes a new set of dies, drive up close and try it in the mouth, it generally will fit, if not, blames it on the *impression* and takes a new one, and proceeds as before, but with greater care. Rarely uses chambers, but places one or two thicknesses of paper over the hard palate to prevent rocking, try it in the mouth, arrange the teeth *just right*, grind the ends of the gums off square, so as to leave them stong, take a strip of plate, and solder on so as to cover the ends of the teeth (not lap over) but barely cover the ends, thus solders on another strip, which makes a double plate as a base, or rest for the ends of the gum, objects to the rim which laps over the ends of the teeth, as it forms a trough or cavity which it is impossible to keep perfectly clean. Now place within a German silver rim made for the purpose, and invest in plaster, put on the backings, uses double backings fitted to the teeth and dressed first, if teeth are irregular in length must make the backs especially adapted for each tooth, fit them into place and dress the backs rounding, put the teeth back to their place, put on some wax, remove the plaster and apply fresh plaster. He uses solder which melts but little, below the plate—for soldering to the plate, uses solder that flows a little easier. Solders all kinds of work with a mouth-pipe, it requires from ten to fifteen minutes to solder a full upper set, has done it in eight minutes and has put an entire job through from 9 A. M, to 6 P. M. If there should happen to be any crevice between the tooth and plaster, melt some clean white wax and pour it in, this keeps the work clean and sweet.

Dr. Chittenden expressed great gratification, had used the old rim, coming over the ends of the teeth, which he always deemed a sinkhole of filth. This mode of Dr. B. is "just the thing," is glad he come, and feels well paid for the trip, etc.

Dr. Porter differs in removing the rim although it amounts to the same—he pours plaster *inside*, a little over the edges of the teeth, thus retains them constantly in position, and is enabled to take them off and put them on at pleasure.

Dr. Field has done some cheoplastic work, but don't think much of it, uses also gold plate-work and continuous gum-work, the latter he thinks the best. Gets up his plates as before described, but uses a *double* plate, the inner one reaching just to the alveolar ridge and terminating in the upturned flange or rim under and against which the "body" is packed and flowed, rims inside and outside, thus giving great strength—never heard any complaint of weight. If a plate fits accurately, the weight is imperceptible. Uses Robert's Gum, never had a job to crack, scale or break, except from falling. If the furnace is hot, can mend or replace a tooth or teeth in half a day. Grind out to admit the tooth, put on a back and solder to the old back, build up with body and gum and bake as a new piece, no scar or imperfection can be seen, in appearance the mending can not be perceived.

Dr. Bancroft likes cheoplasty, *Blandy's Cheoplasty*, don't know anything about any other, has supplied cheoplasty to patients who had been wearing gold, and *they say* it tarnished less than gold, knows of several cases doing remarkably well.

Dr. Chittenden uses cast plates in the lower jaw on account of the weight, which he deems to be an advantage in many cases.

The subject of "*Solder*" was now opened.

Dr. Farmer uses no zinc in solder, but takes scraps of gold plate and adds silver and copper. In cheoplasty the fit is so perfect the weight is inappreciable, if it weighed three ounces it would not be observed. Had a case in which the muscles were trouble *some*, cut the muscles away and now has no trouble, and the lady has remarked she "would not take *two gold* plates for it." Makes his own compound.

On motion of Dr. Chittenden, a committee of three was appointed to prepare the business and a list of subjects for discussion at the next meeting.

Drs. Porter, Chittenden and Whiting were appointed the committee, with instructions to publish in the DENTAL REPORTER, not later than the August Number.

Motion of Dr. Benedict that each member is hereby earnestly requested to prepare an article or essay in writing, on one of the subjects to be discussed, to be delivered in person, or sent in by letter, and read before the society, also that all come prepared with written notes, of the heads or points for remark on the different subjects. Adopted.

Motion of Dr. Benedict that this society when it adjourns, will do so to meet again on the second Tuesday in January, 1860, in the city of Detroit. Adopted.

Motion of Dr. Bancroft that thanks of this Association be tendered to Drs. Whiting and Benedict, for the use of their rooms, occupied by the Association.

Motion of Dr. Porter that the thanks of the Association be tendered to Mr. Toland for his attendance and assistance, with an urgent hope that he will favor us with his presence at our next meeting. Adopted.

Being loudly called for, it was deemed necessary to make a response, which our *modesty* confined to an acknowledgment of the kindness and honors of which we were the recipient.

Motion of Dr. Farmer that a list of those present, and a brief sketch of these proceedings be published in the daily and weekly papers of this city.

At ten o'clock the Society adjourned, to meet next year at same place, of which ample notice will be given.

---

CINCINNATI, O., Jan. 25, 1859.

FRIEND TOLAND:—In the last number of the REPORTER, you gave notice of the formation of our Local Association and its proceedings up to that time. Please note now that it still lives, and with life gains new strength and vigor, and we confidently hope to boast of an organization which shall soon be without a rival. Such at least is our ambition, and I hope it will not be considered presumption in me to say we have the material to work upon and accomplish that end. We have held regular monthly meetings, and can assure our brethren in the East, West, North and South, that we will not leave a "stone unturned" for the promotion or advancement of our science. We wish to attract their attention because it may *possibly* be of some benefit to them, and I know it will be to us. One of our brethren in the west appears somewhat solicitous about the government of our association; be assured, kind sir, that our standard is high, and we will have a government, *second* to none. You may at some future day, be furnished with a copy of our Constitution and *By-laws*, also a list of the names of members. Wishing yourself and all our *professional* brethren in the Union, the greatest success and many happy New-Years,

I remain yours, &c., JUNIUS.

